# A comparative effectiveness analysis of the PBCG vs. PCPT risks calculators in a multi-ethnic cohort

**DOI:** 10.1186/s12894-019-0553-6

**Published:** 2019-11-27

**Authors:** Samuel Carbunaru, Oluwarotimi S. Nettey, Pooja Gogana, Irene B. Helenowski, Borko Jovanovic, Maria Ruden, Courtney M. P. Hollowell, Roohollah Sharifi, Rick A. Kittles, Edward Schaeffer, Peter Gann, Adam B. Murphy

**Affiliations:** 1 Department of Urology, Northwestern University Feinberg School of Medicine, 303 E Chicago Avenue, Tarry 16, Chicago, IL 60611 USA; 2 Department of Preventive Medicine, Northwestern University Feinberg School of Medicine, Chicago, IL USA; 3 Department of Medicine, University of Illinois at Chicago, Chicago, IL USA; 4 Division of Urology, Cook County Health and Hospitals System, 303 E Chicago Avenue, Tarry 16, Chicago, IL 60611 USA; 5 Section of Urology, Jesse Brown VA Medical Center, 303 E Chicago Avenue, Tarry 16, Chicago, IL 60611 USA; 6 Department of Urology, University of Illinois at Chicago School of Medicine, Chicago, IL USA; 7 Division of Health Equities, Department of Population Sciences, City of Hope Cancer Center, Duarte, CA USA; 8 Department of Pathology, University of Illinois at Chicago School of Medicine, Chicago, IL USA

**Keywords:** Prostate biopsy collaborative group risk calculator, Prostate Cancer prevention trial risk calculator 2.0, Prostate cancer risk prediction, Risk calculator, African American validation

## Abstract

**Background:**

Predictive models that take race into account like the Prostate Cancer Prevention Trial Risk Calculator 2.0 (PCPT RC) and the new Prostate Biopsy Collaborative Group (PBCG) RC have been developed to equitably mitigate the overdiagnosis of prostate specific antigen (PSA) screening. Few studies have compared the performance of both calculators across racial groups.

**Methods:**

From 1485 prospectively recruited participants, 954 men were identified undergoing initial prostate biopsy for abnormal PSA or digital rectal examination in five Chicago hospitals between 2009 and 2014. Discrimination, calibration, and frequency of avoided biopsies were calculated to assess the performance of both risk calculators.

**Results:**

Of 954 participants, 463 (48.5%) were Black, 355 (37.2%) were White, and 136 (14.2%) identified as Other. Biopsy results were as follows: 310 (32.5%) exhibited no cancer, 323 (33.9%) indolent prostate cancer, and 321 (33.6%) clinically significant prostate cancer (csPCa). Differences in area under the curve (AUC)s for the detection of csPCa between PCPT and PBCG were not statistically different across all racial groups. PBCG did not improve calibration plots in Blacks and Others, as it showed higher levels of overprediction at most risk thresholds. PCPT led to an increased number of avoidable biopsies in minorities compared to PBCG at the 30% threshold (68% vs. 28% of all patients) with roughly similar rates of missed csPCa (23% vs. 20%).

**Conclusion:**

Significant improvements were noticed in PBCG’s calibrations and net benefits in Whites compared to PCPT. Since PBCG’s improvements in Blacks are disputable and potentially biases a greater number of low risk Black and Other men towards unnecessary biopsies, PCPT may lead to better biopsy decisions in racial minority groups. Further comparisons of commonly used risk calculators across racial groups is warranted to minimize excessive biopsies and overdiagnosis in ethnic minorities.

## Background

Prostate cancer (PCa) is the leading malignancy amongst men with 164,690 new diagnoses in the United States in 2018 [1]. Prostate specific antigen (PSA) screening has reduced PCa specific mortality by ~ 50% [2]. Approximately 1 million biopsies are performed in the US each year, however, 54% of them are negative and another 25% reveal presumably indolent PCa [3, 4]. Complications of prostate biopsy are not uncommon and should be considered. Infectious complications affect 0.1 to 7.0% of patients, followed by sepsis ranging from 0.3 to 3.1% [5]. To mitigate screening harms, several predictive risk calculators (RCs) have been developed to help men make informed decisions about biopsy and better identify men who are likely to have clinically significant PCa. However, it is unclear if commonly used RCs are appropriately calibrated to identify racial minorities who can avoid unnecessary biopsies. While racial disparities in PCa between US Blacks and Whites have diminished over the past decades, there are still considerable differences [6]. Black men present with higher PSA levels, are at higher risk of developing clinically significant PCa (csPCa), and have higher mortality rates compared to Whites [7, 8]. The commonly used Prostate Cancer Prevention Trial 2.0 (PCPT) and the new Prostate Biopsy Collaborative Group (PBCG) RCs both take race into consideration, but were developed in largely European ancestry populations [9, 10].

This study compares the PBCG and PCPT RCs across racial groups using discrimination and calibration statistics, as well as the frequency of avoided biopsies and missed csPCa in an urban, multi-racial cohort.

## Methods

### Study participants

Following institutional review board approval at Northwestern University, University of Chicago, University of Illinois at Chicago, Jesse Brown VA Medical Center and Cook County Health from 2009 to 2014, 954 consecutive ambulatory men from urology clinics at two privately funded and three publicly funded institutions were enrolled in a cross-sectional study evaluating the association between vitamin D status and prostate biopsy outcomes [11]. Patients were deemed eligible if they were undergoing their first prostate biopsy for an abnormal PSA level or digital rectal exam (DRE). All patients provided written informed consent.

### Data collection

A self-administered questionnaire confirmed self-reported race & ethnicity, demographics, and medical history. Histologic diagnosis, DRE, and imaging reports were evaluated to determine disease stage according to American Joint Committee on Cancer TNM (tumor, node, metastasis) staging system [2]. All patients underwent a transrectal ultrasound guided biopsy with at least a 10-core biopsy with a median of 12 cores. Biopsies were read by three experienced uropathologists at Northwestern (XY) and at the University of Illinois at Chicago (ABJ and VM).

### Statistical analysis

Descriptive statistics were used to characterize important covariates including age, race, PSA, PSA density, prostate volume, body mass index (BMI), alcohol and smoking use, income, family history of PCa, marital status, abnormal DRE, college completion, 5 alpha-reductase inhibitor (5-ARI) use, and the clinical diagnosis of benign prostatic hyperplasia (BPH). To compare groups, Student’s *t*-tests or nonparametric Wilcoxon-Mann tests were performed for continuous variables, and Pearson-*χ*^2^ tests were used for categorical variables.

The PCPT RC 2.0 [10] and PBCG [9] were applied, as provided in R package, to estimate the risk of overall PCa and csPCa (defined as Gleason ≥3 + 4) for each participant using PSA, DRE, first-degree family history of PCa (father, brother or son ever diagnosed with PCa) and history of a prior negative prostate biopsy. All patients had no prior prostate biopsy as they were recruited at time of initial biopsy. Categories for self-reported race included Black/African American, White/Caucasian, Hispanic and Other. Because published logistic regression coefficients in the PCPT RC [10] computed probabilities by race similarly for ‘Hispanic’ and ‘Other’, we categorized all participants who were non-Black and non-White as ‘Other’ in our analysis. For participants with unknown family history or DRE status, “Do not know” and “Not performed or not sure”, respectively, were used following the recommendation by the online RCs. Percent free PSA, PCA3, and TMPRSS ERG were not included in the risk calculations since the test was not routinely ordered for all participants. The primary endpoints were the presence of any prostate adenocarcinoma and the presence of Gleason ≥3 + 4 PCa on prostate biopsy. Of the Gleason 6 tumors, 77% were very low to low risk according to NCCN guidelines [12].

Discrimination was calculated by quantifying the nonparametric area under the receiver operating characteristics curve (AUC). AUC was calculated for PBCG and PCPT by race (Black, White, Other). We were powered to detect a 7% AUC difference between PCPT and PBCG in Blacks and Whites at alpha = 5% with greater than 99% power. For Other men we achieved a 76% power. We assumed a PCPT AUC of 0.60 with a one-sided alpha of 0.05, and a 0.95 correlation between PCPT and PBCG for both positive and negative results.

Calibration curves were generated by plotting the predictions generated by the PBCG and PCPT RC on the *x*-axis in deciles and the observed outcomes for men in that decile on the *y*-axis. In the calibration plot, the 45-degree line represents perfectly calibrated predictions. A Hosmer-Lemeshow test for goodness-of-fit was performed to assess the quality of the calibration of each risk calculator.

Decision curve analysis (DCA) is a graphical statistical method that plots a population’s net benefit from a RC on the *y*-axis over a variety of probabilities for the calculator detecting true disease on the *x*-axis [13]. Patients and providers usually have individual *probability thresholds* of disease detection above which they would undergo or recommend the biopsy that range between 5 and 40% [14–17]. The probability threshold can be used to estimate how a decision maker might weigh the relative benefit of appropriate treatment compared to the potential harms of undergoing unnecessary biopsy. The net benefit of PBCG and PCPT has not been compared across race previously.

The net benefits were compared using the following biopsy strategies: Biopsy All men, Biopsy based on PBCG Thresholds, and Biopsy based on PCPT Thresholds. For the Biopsy All men curve, an exchange rate of 1/9 was used (as in Vickers et al.’s analysis of PCa biomarkers [18]), meaning we are willing to perform nine unnecessary biopsies to detect one case of csPCa.

Statistical analyses were performed using Excel, SPSS 24 (IBM Corporation 2016, United States), R 3.3.3., SAS v9.4 (SAS Institute, Cary, NC, USA), and Stata 12.1 (StataCorp, 2011, College Station, TX).

## Results

### Demographics

In total, 954 men with elevated PSA levels or abnormal DRE results underwent an initial transrectal ultrasound guided prostate biopsy during 2009–2014 (see Table 1). The sample included men who self-reported as Black (463, 48.5%), White (355, 37.2%), and Other races (136, 14.2%). The Other racial group included Hispanic (*n* = 103, 75.7%), Asian (*n* = 28) and Middle Eastern men (*n* = 5). All groups had a comparable age (*p* = 0.47) and BMI (*p* = 0.56). Black men had higher pre-biopsy PSA values and density (both *p* < 0.001).

**Table 1:** Patient sociodemographic characteristics and clinical risk factors by race

Continuous Variables	Black (*n* = 463)	White (*n* = 355)	Other (*n* = 136)	*p* Value ^1^
	Median [IQR]	Median [IQR]	Median [IQR]	
Age, years	61.0 [57.0, 67.0]	62.0 [58.0, 67.0]	62.0 [57.0, 67.0]	0.47
BMI, kg/m ^2^	27.8 [24.5, 31.8]	27.5 [25.0, 30.6]	27.4 [24.2, 30.3]	0.56
PSA, ng/ml	7.8 [5.3, 15.5]	4.8 [3.6, 7.3]	7.2 [5.1, 12.4]	**< 0.001**
Prostate Volume, cm ^3^	39.5 [28.8, 55.4]	39.5 [29.7, 51.0]	48.7 [30.0, 69.4]	**< 0.001**
PSA density, ng/cm^3^	0.23 [0.12,0.43]	0.13 [0.09, 0.20]	0.18 [0.11, 0.32]	**< 0.001**
PCPT csPCa Risk, %	25.0 [17.0, 40.0]	7.0 [5.0, 12.0]	11.5 [7.0, 18.0]	**< 0.001**
PBCG csPCa Risk, %	45.0 [31.0, 65.0]	26.0 [18.0, 41.0]	37.5 [22.0, 54.2]	**< 0.001**
Categorical Variables	**Black n (%)**	**White n (%)**	**Other n (%)**	***p*** **Value**^**2**^
Clinical Risk Factors				
Family History of PCa	93 (20.1)	88 (24.8)	21 (15.4)	0.055
Abnormal DRE	140 (30.3)	112 (31.5)	43 (31.3)	0.92
BPH/LUTS	151 (32.6)	135 (38.0)	66 (48.5)	**0.003**
5-ARI Use (current)	43 (9.3)	19 (5.4)	26 (19.1)	**< 0.001**
Substance Use History				
Heavy Smoking (>1ppd)	29 (6.4)	19 (5.4)	1 (0.7)	**0.034**
Heavy Alcohol Use (> 10 drinks/week)	30 (6.6)	46 (13.0)	5 (3.7)	**< 0.001**
Demographic Factors				
Married	192 (42.1)	269 (76.2)	105 (77.2)	**< 0.001**
College Completion	165 (36.2)	248 (70.3)	46 (33.8)	**< 0.001**
Income < $30,000	161 (43.9)	39 (12.0)	42 (55.2)	**< 0.001**
Biopsy Outcomes				**< 0.001**^3^
Indolent Cancer	167 (36.1)	118 (33.2)	38 (27.9)	0.20
Clinically Significant Cancer	175 (37.8)	120 (33.8)	26 (19.1)	**< 0.001**
No Cancer	121 (26.1)	117 (33.0)	72 (52.9)	**< 0.001**

^1^Using Kruskal-Wallis tests; ^2^Using Pearson-χ2 tests; ^3^χ2 trend test. Bold type indicates *p* values < 0.05. Abbreviations: *BMI* body mass Index, *PSA* prostate specific antigen, *BPH/LUTS* clinical diagnosis of Benign Prostatic Hyperplasia/Lower Urinary Tract Symptoms, *DRE* digital rectal examination, *csPCa* clinically significant prostate cancer, *PBCG* Prostate Biopsy Collaborative Group, *PCPT* Prostate Cancer Prevention Trial, *ppd* pack per day

Of the 954 biopsies, 310 (32.5%) were negative for PCa, 323 (33.9%) were positive for indolent PCa, and 321 (33.6%) demonstrated csPCa. Black men were more often diagnosed with overall PCa (73.9%) and csPCa (37.8%) on biopsy than White or Other men (both *p* < 0.001). A comparison of median risk scores shows that Blacks had higher risk scores for csPCa compared to White and Other men in both PCPT and PBCG (p < 0.001).

### Discrimination/calibration

Statistically, there was no difference in the AUCs between PBCG and PCPT for overall PCa when all men are included. The AUC for csPCa was 0.64 (95% CI: 0.61–0.68) for PCPT and 0.65 (95% CI: 0.62–0.68) for PBCG (*p* = 0.27). The AUCs slightly improved with PBCG in Whites (0.64 vs. 0.66; *p* = 0.07) and Blacks (0.67 vs. 0.68; *p* = 0.25), but not in Others (0.64 vs. 0.64; *p* = 0.81); yet, none of these differences were statistically significant.

**Fig. 1:**
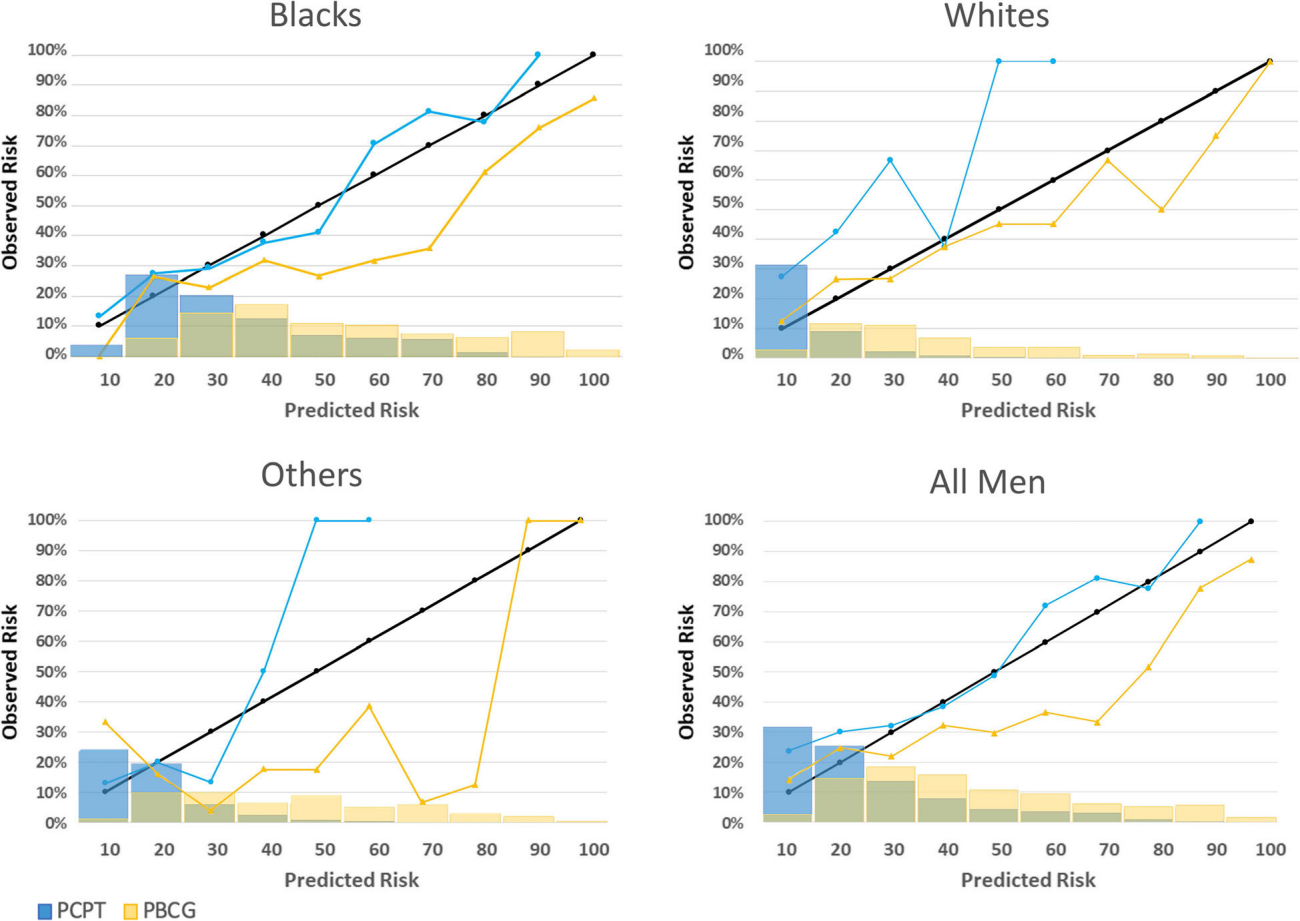
Race-stratified calibration curves and the risk probability distribution histograms for clinically significant prostate cancer for PBCG and PCPT risk calculators. PCPT = Prostate Cancer Prevention Trial risk calculator; PBCG = Prostate Biopsy Collaborative Group risk calculator; Blue lines and histograms = PCPT; Yellow lines and histograms = PBCG; Black lines = 45-degree line representing perfect calibration

Figure 1 depicts the calibration and distribution plots for csPCa by racial group. The PBCG calculator results in a broader distribution of men in all racial groups, as opposed to the PCPT RC where most men are clustered in the lower risk deciles (< 30%). The PBCG calibration in all men outperforms PCPT at the < 30% range, while the PCPT is better calibrated at ≥30% risk deciles. It is unclear if the improved calibration at probabilities ≥30% is clinically significant since most men will opt for biopsy at > 30% risk [16]. For Blacks and Others PCPT seems to be better calibrated, as PBCG over-predicts csPCa across most risk thresholds. In Whites, however, the opposite is seen with PBCG being better calibrated, since PCPT underestimates the risk of csPCa in the 10–60% range. After performing a Hosmer-Lemeshow test for goodness-of-fit, no statistically significant changes were detected in calibration plots between PCPT and PBCG in any racial group: Blacks (*p* = 0.15), Whites (*p* = 0.08), and Others (*p* = 0.07).

### Number of avoided biopsies and missed clinically significant prostate cancer

**Fig. 2:**
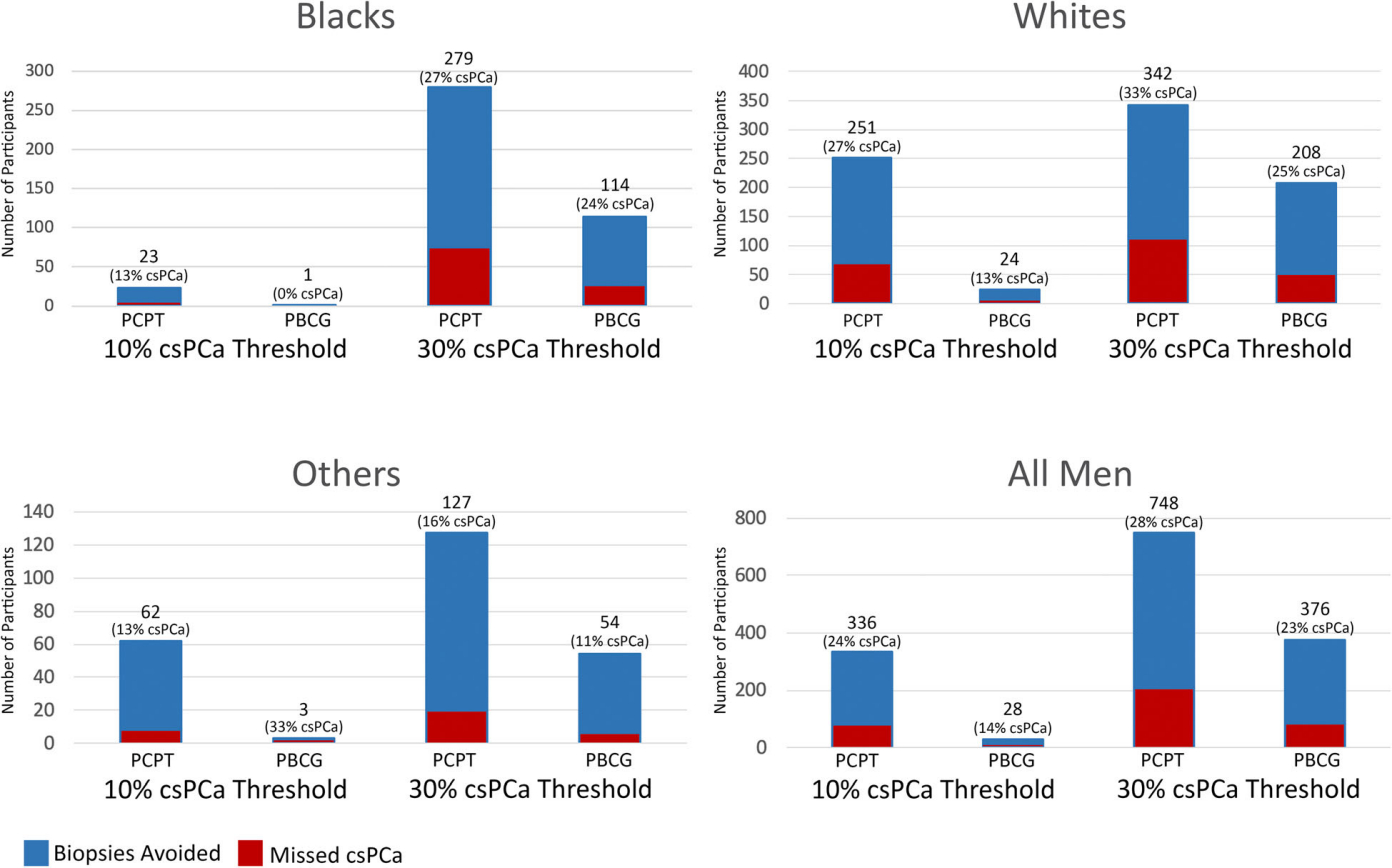
Theoretical number of avoided biopsies and missed clinically significant cancers at the 10 and 30% predictive thresholds by race. PCPT = Prostate Cancer Prevention Trial risk calculator; PBCG = Prostate Biopsy Collaborative Group risk calculator; csPCa = clinically significant prostate cancer. Blue bar: Total number of biopsies avoided; Red bar: Missed clinically significant prostate cancer

Figure 2 presents the theoretical number of biopsies avoided and missed csPCa at the ≥10% and ≥ 30% risk thresholds.

At the ≥10% threshold, assuming no biopsies are performed below this threshold, the number of biopsies avoided with the PCPT RC for All men is 336/954 (35%) compared to 28/954 (3%) with PBCG. The PCPT RC, however, missed 80 (24%) csPCas compared to 4 (14%) csPCas when using PBCG. Few Black men fall below the 10% risk threshold, so the number of avoided biopsies is small with both calculators. The difference is particularly prominent in Whites where the percentage of biopsies avoided is ten-fold higher with PCPT relative to PBCG (71% vs 7%).

At the ≥30% threshold in All men, 748 (78%) biopsies are avoided using PCPT compared to 376 (39%) with PBCG, and the number of missed csPCa is 207 (28%) and 85 (23%), respectively. Black and Other men demonstrate a similar trend, where more than twice as many biopsies are avoided using PCPT with similar rates of missed csPCa (27% vs. 24% in Blacks; and 16% vs. 11% in Others).

### Unnecessary biopsies in low risk men

The proportion of low risk men (i.e. men with PSA < 10 ng/mL and either Gleason 6 tumors or no cancer) who underwent unnecessary biopsies was assessed by racial group. At a threshold of ≥10%, assuming that men with higher scores are biopsied, 250/487 (51%) low risk men would have undergone a biopsy with PCPT and 466 (96%) with PBCG. Almost all low risk Black men are biopsied with both PCPT (92%) and PBCG (99.5%). For Whites and Others, the proportion of low risk men biopsied with PCPT is much lower relative to PBCG (see Additional file 1).

At the ≥30% threshold, PCPT would spare most low risk men a biopsy and only subject 5% to a prostate biopsy, while 42% are still biopsied with PBCG. In Blacks, the number of low risk men biopsied substantially decreases to 25 (12%) with PCPT, but continues to remain high with PBCG at 121 (59%). There were no White and Other men biopsied with PCPT, while 27 and 38% were biopsied using PBCG, respectively. The increase in risk scores seen in PBCG does not spare low risk men, resulting in many unnecessary biopsies performed in men with indolent or no PCa.

### Decision curve analysis: net benefit

**Fig. 3:**
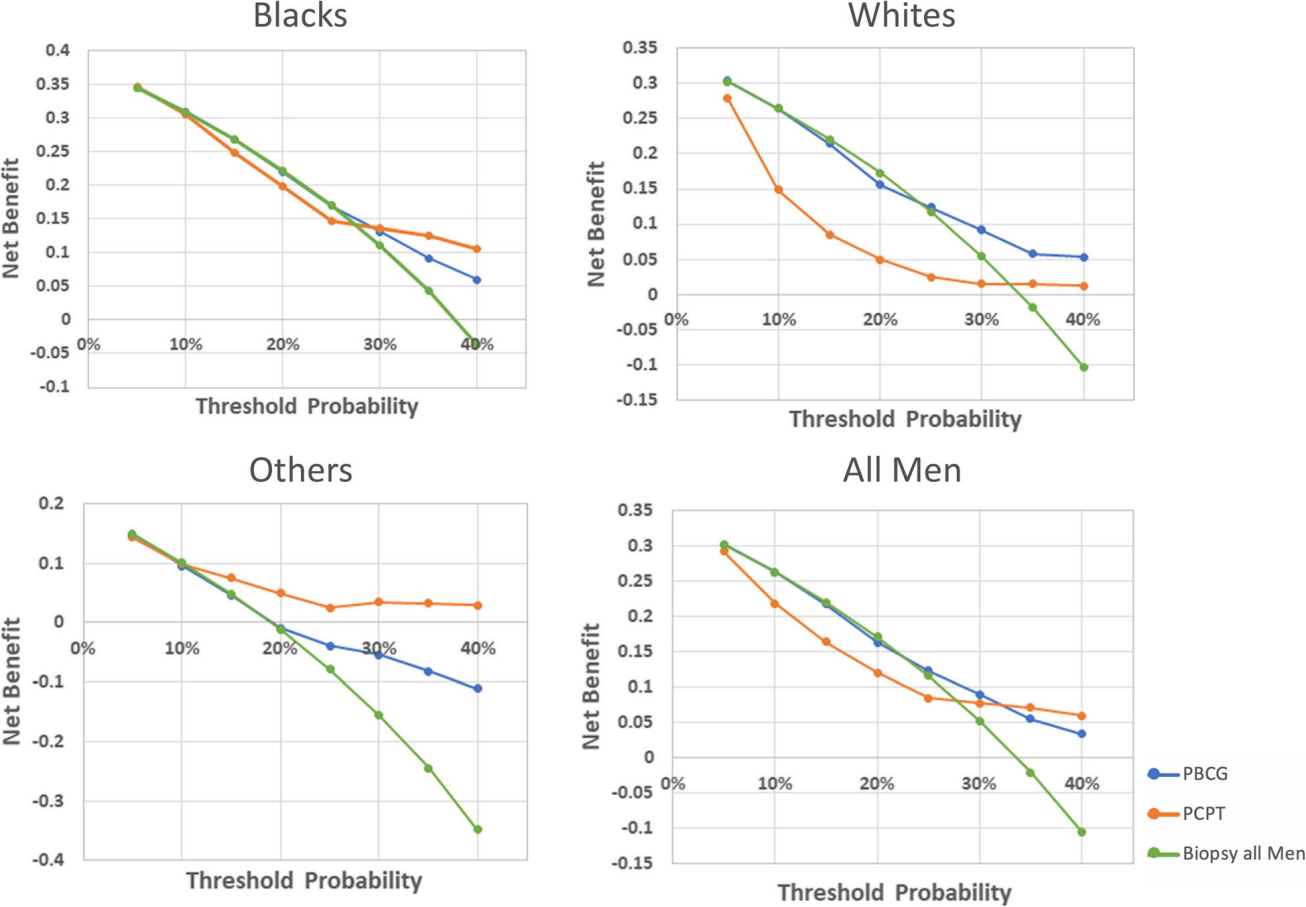
Net Benefit curves for clinically significant prostate cancer prediction comparing PBCG to PCPT and the Biopsy all Men strategy by race. PCPT = Prostate Cancer Prevention Trial risk calculator; PBCG = Prostate Biopsy Collaborative Group risk calculator. Green line = Biopsy All men (assuming an exchange rate = 1/9); Orange line = Biopsy based on the probability risk thresholds of the PCPT risk calculator; Blue line = Biopsy based on the probability risk thresholds of the PBCG risk calculator

The net benefit of each risk model is displayed graphically in Fig. 3. When calculating the net benefit analysis for All men, we note that the net benefit for PBCG is higher than PCPT at low threshold probabilities; however, neither RC shows higher net benefit than the Biopsy All men strategy at thresholds below 25%. At the higher risk thresholds (> 30%), PCPT surpasses the net benefit of PBCG.

These results differ vastly by race. Black men show similar trends as the ones described above with PBCG having higher net benefits than PCPT at lower thresholds, but not at thresholds above 30%. White men show a more pronounced improvement with PBCG across all thresholds. In Other men, the opposite is seen with PCPT showing higher net benefit than PBCG throughout all thresholds. Overall, PBCG demonstrates improved net benefits below the 30% threshold in Whites and, to lesser extent, in Blacks, but not in Others.

## Discussion

Discrimination between PBCG and PCPT was not statistically different for overall or csPCa (*p* = 0.27). Although Ankerst et al. did show a statistically significant 3% improvement in PBCG over PCPT on both the internal and external validation [9], our study was not powered to detect this difference. The PCPT RC has been mostly validated in populations of European descent, which might not be representative of the demographics in the United States. The PCPT 2.0 risk calculator development cohort included 219 (3.3%) Blacks, but did not report the AUCs for csPCa by race. This study includes a multi-racial cohort of men recruited from five institutions in a large metropolitan city. Few studies have evaluated the performance of the PCPT RC in diverse populations. The Durham VA (North Carolina) cohort enrolled high numbers of Black men (45%) and demonstrated an AUC of 0.74 [19]. The Cleveland Clinic cohort was composed of 13% Black men and had an AUC of 0.64 [19]. Lastly, the SABOR Cohort from San Antonio, Texas compared PCPT across racial groups and showed that PCPT performs best in Black men compared to other races (AUC 0.80 vs. 0.66, *p* = 0.02) [20]. The AUC for Blacks in our study (0.67) was notably lower than SABOR’s and Durham’s, and similar to the Cleveland Clinic’s cohort. Like SABOR’s, the AUC for Blacks in our cohort was slightly higher than Whites’ (0.64), although this was not statistically significant.

In terms of the calibration curves, PBCG’s calibrations in White men appear to be superior to PCPT. Blacks and Others show a different trend, where PCPT appears better calibrated since PBCG overestimates the rates of csPCa. Ankerst et al.’s validations also show a widened distribution for PBCG across all thresholds [9]. Their results are similar to the calibration plots we obtained for csPCa in our White population, but not in our minority groups. Since their internal and external validations had a non-Black population of 87% and 99.7%, their results come from a more racially homogeneous sample [9], which might explain these discrepancies. To our knowledge, no other studies have validated PBCG in a racially diverse cohort.

The cutoff most urologists use to decide if a patient should undergo a biopsy lies somewhere between the 5–30% threshold [18]. Therefore, this range is where nomograms serve the greatest clinical utility. At the 30% threshold, PCPT results in more than twice the amount of avoided biopsies with similar rates of missed csPCa in Blacks and Others (see Fig. 2). In addition, despite similar frequencies of indolent PCa and negative biopsy in Blacks and Whites (62.2% vs. 66.2%, *p* = 0.24), PBCG disproportionally biases more low risk Black men towards unnecessary biopsies (59% low risk men missed) relative to White men (27% missed).

The net benefits and clinical utility of different strategies vary widely by race, as seen in Fig. 3. In our data, White men and Black men seem to have higher net benefit with PBCG at relevant thresholds between 0 and 25%. For Others, PCPT has higher net benefit across all thresholds, which might be due their lower prevalence of csPCa compared to Blacks and Whites. Below the 30% threshold, the PBCG RC shows no greater benefit than the “Biopsy All strategy” in Black and White men, indicating that the conservative cutoff of 10% might not have any clinical benefit to avoid unnecessary biopsies.

Our comparison of PBCG vs. PCPT concurs with the study published by Ankerst et al. in that PBCG has higher discrimination [9], though not statistically significant in our smaller sample. However, we find that the performance of both calculators varies widely by race. Unfortunately, Ankerst et al. did not include an analysis on how the calibration or net benefit plots differ by racial groups. Although the PBCG RC was developed in a cohort with 13% Blacks, the validation study only included 33 (0.3%) Blacks in the cohort. Our results coincide with Ankerst et al. in that PBCG results in significant improvements compared to PCPT in Whites; yet, we find that the PBCG RC results in overprediction of PCa and a significant increase in the number of unnecessary biopsies in racial minorities. Given that PCPT was of higher accuracy in Blacks in prior validations, it may be prudent to continue to use it in biopsy decision making for Blacks. This should be compared in future studies using other diverse cohorts.

With the increasing popularity of multiparametric magnetic resonance imaging (mpMRI) as a PCa detection method, many are doubting the current need for risk calculators or are incorporating mpMRI PIRADS scores into them [21]. There is substantial evidence supporting the advantage of mpMRI, as it has been shown to avoid biopsies in around 28% of men and subsequently decrease the overdiagnoses of indolent PCa [15]. However, the adoption of mpMRI would add approximately $3 billion annually, meaning that this diagnostic test would account for 15% of all PCa related cost [22]. The use of mpMRI has become common practice in large academic institutions; however, 70% of community hospitals have not adopted such practice and 75% of hospitals perform few mpMRI (< 20 mpMRI/month) [22]. Currently, a patient’s geographic location (non-urban settings) and insurance type (health maintenance organizations) greatly reduce their chances of having access to a mpMRI [22, 23]. The accuracy of mpMRI in community settings is also risky with only 55% concordance between community and expert academic radiologists [24]. While mpMRIs show promising results, the need for predictive nomograms is still warranted in areas of the country that face barriers in the implementation of mpMRIs.

This study has several limitations that should be noted. The recruitment was done in tertiary and publicly funded medical centers in a large metropolitan city. Our population was recruited from the outpatient Urology clinics between 2009 and 2014 and Gleason grading has shifted several Gleason 6’s to Gleason 7 tumors limiting generalizability to our contemporary patients [25]. Our Gleason 6 tumors were consistent with NCCN very low or low risk group in 77% of cases. There were low numbers of non-Black minorities enrolled, which limits power and prevents sub-group analyses of Hispanics, Asians and other ethnic groups. The PCPT RC can accommodate biomarkers that we did not account for like PCA3, free PSA, and TMPRSS2–ERG [26].

## Conclusions

Since PBCG’s improvements in Blacks are disputable and potentially biases a greater number of low risk Black and Other men towards unnecessary biopsies, PCPT may lead to better biopsy decisions in racial minority groups. Further validation studies in racially diverse cohorts are warranted to mitigate the harms of PSA screening in ethnic minorities.

## Supplementary information

**Additional file 1. **Percentage of low risk men who underwent an unnecessary prostate biopsy at the 10 and 30% risk thresholds stratified by race. Low risk Men includes men with PSA < 10 ng/mL and either a negative biopsy (*n* = 238) or Gleason 3 + 3 prostate cancer and less than clinical T3a (*n* = 249); PCPT = Prostate Cancer Prevention Trial Risk Calculator; PBCG = Prostate Biopsy Collaborative Group Risk Calculator.

## Data Availability

The datasets used and/or analyzed during the current study are available from the corresponding author on reasonable request.

## Abbreviations

5-ARI: 5 alpha-reductase inhibitor
AUC: Area Under the Receiver Operating Characteristics Curve
BMI: Body Mass Index
BPH: Benign Prostatic Hyperplasia
csPCa: Clinically Significant Prostate Cancer
DCA: Decision Curve Analysis
DRE: Digital Rectal Exam
PBCG: Prostate Biopsy Collaborative Group
PCa: Prostate Cancer
PCPT: Prostate Cancer Prevention Trial 2.0
PSA: Prostate Specific Antigen
RC: Risk Calculators
