# Supplementary material for: A comparative effectiveness analysis of the PBCG vs. PCPT risks calculators in a multi-ethnic cohort
Source: BMC Urol. 2019 Nov 27;19:121. doi: 10.1186/s12894-019-0553-6 (PMC6880480; doi:10.1186/s12894-019-0553-6)
Supplement: Supplementary file 1 — Additional file 1. Percentage of low risk men who underwent an unnecessary prostate biopsy at the 10 and 30% risk thresholds stratified by race. Low risk Men includes men with PSA < 10 ng/mL and either a negative biopsy (n = 238) or Gleason 3 + 3 prostate cancer and less than clinical T3a (n = 249); PCPT = Prostate Cancer Prevention Trial Risk Calculator; PBCG = Prostate Biopsy Collaborative Group Risk Calculator. [file 12894_2019_553_MOESM1_ESM.docx]

Supplementary Table 1. Percentage of low risk men who underwent an unnecessary prostate biopsy at the 10% and 30% risk thresholds stratified by race.

Note: Low risk Men includes men with PSA<10ng/mL and either a negative biopsy (n = 238) or Gleason 3+3 prostate cancer and less than clinical T3a (n = 249); PCPT = Prostate Cancer Prevention Trial Risk Calculator; PBCG = Prostate Biopsy Collaborative Group Risk Calculator.

|  | **Low Risk Men Biopsied** | | | |
| --- | --- | --- | --- | --- |
|  | ≥**10%** | | ≥**30%** | |
| **Total Low Risk Men** | **PCPT** | **PBCG** | **PCPT** | **PBCG** |
| Black  n = 205 | 189 (92%) | 204 (99.5%) | 25 (12%) | 121 (59%) |
| White  n = 209 | 38 (18%) | 191 (91%) | 0 (0.0%) | 57 (27%) |
| Other  n = 73 | 23 (32%) | 71 (97%) | 0 (0.0%) | 28 (38%) |
| All  n = 487 | 250 (51%) | 466 (96%) | 25 (5%) | 206 (42%) |
